# Posttraumatic Pedal Artery Pseudoaneurysm: A Case Report

**DOI:** 10.1155/2012/234351

**Published:** 2012-03-29

**Authors:** Cristián Arriagada Irarrazaval, Ricardo Sonneborn Gross, Alexandre Sauré Maritano, Carolina Soto Diez

**Affiliations:** ^1^Burns Surgery Unit, Hospital Asistencia Publica, Santiago, Chile; ^2^Avenida Presidente Kennedy 5334, Apartament 51, Vitacura, 7630586 Santiago, Chile; ^3^Surgery Department, Hospital del Trabajador, Santiago, Chile

## Abstract

Pseudoaneurysm of the pedal artery is usually caused by traumatic or iatrogenic events. Diagnosis is suspected by palpation of a pulsatile mass and detection of an associated systolic bruit. Confirmation is made by Doppler US. Angiography can demonstrate the presence of collateral circulation and assure distal vascular flow. We present the case of a 49-year-old man who presents 3 months after a traumatic contusion of his left foot with a pulsatile mass. A color Doppler ultrasound confirms a pseudoaneurysm of the pedal artery. The patient underwent surgical resection of the mass and suture ligation of the artery with full recovery and no ischemic complications.

## 1. Introduction

Arterial pseudoaneurisms consist in a dilation of an artery with actual disruption of one or more layers of its walls, rather than with expansion of all wall layers. These formations in the pedal artery are very unusual. They are commonly caused by a traumatic or iatrogenic lesions. Patients usually complain of an enlarging pulsatile mass. Diagnosis can be made by Doppler ultrasound or angiography. The treatment is surgical and can be made by ligation, repair, or reconstruction. This paper describes a posttraumatic pedal artery pseudoaneurism and its surgical management. A review of the literature is also presented. 

## 2. Case Report

**Figure 1:**
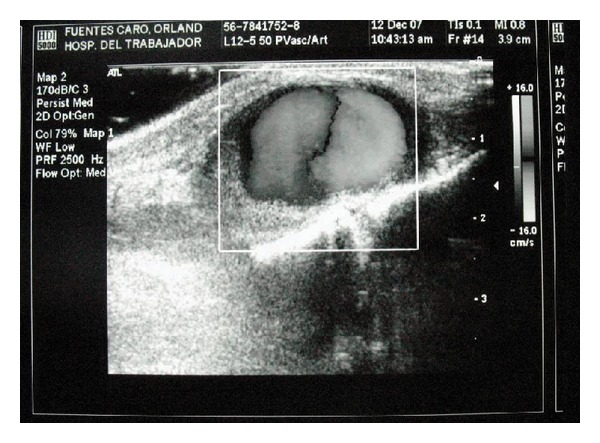
Color Doppler ultrasound demonstrating an hypoechoic 2.64 × 1.53 cms lesion.

**Figure 2:**
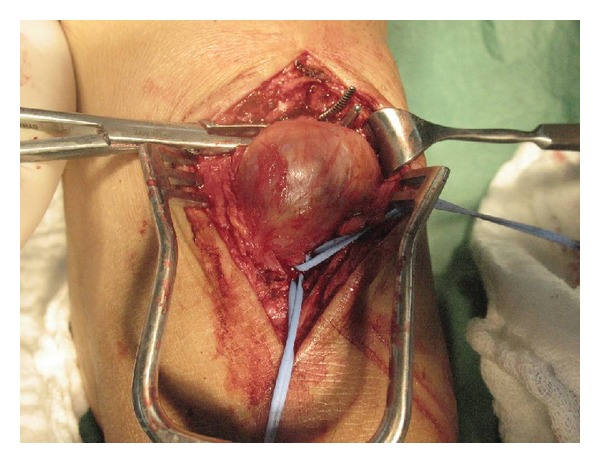
Surgical resection of the pseudoaneurysm.

A previously healthy 49-year-old man presents 3 months after a traumatic contusion of his left foot. Fractures were discarded on an emergency unit after the contusion. He complained of an enlarging mass in the anteromedial surface of his foot. A small mass had appeared a week after the trauma and progressively grew since then. He also referred to moderate pain associated with distal paresthesias in his third and fourth toes. No history or clinical signs of embolization were noted. Clinical examination showed a soft pulsatile mass in the midfoot with a systolic bruit. He had good distal pulses, and the sensorial and motor functions of the foot were conserved. Paresthesias corresponded to the area of the superficial peroneal nerve. A color Doppler ultrasound was performed, and it revealed an hypoechoic 2.64 × 1.53 cms mass dependent on the pedal artery of the left foot (Figure 1). The patient was a construction worker and had serious difficulties using safety footwear, and surgical management was decided due to the functional limitations. Under spinal anesthesia, he underwent aneurysmectomy. The lesion was resected, and the vessels were suture ligated (Figure 2). The foot had good distal flow and no ischemic areas after resection. Anatomopathological study of the tissue demonstrated a pseudoaneurism. The patient recovered uneventfully with no ischemic complications and with full recovery.

## 3. Discussion

Posttraumatic pedal artery pseudoaneurysms are a very rare vascular entity [1, 2]. A literature review was performed on Pubmed with the mesh terms false aneurysm and arteries, (pedal artery). Crossed references were revised. Only 8 cases published between 1978 and 2009 were found. The ages ranged from 6 to 71 years, and the most common causes were nonpenetrating traumatic lesions such as hematomas [3–5], sprains and fractures [6, 7], penetrating trauma [8] as well as iatrogenic lesions secondary to arterial catheterism [9, 10], and surgical amputations [6]. The diagnosis of the pseudoaneurysm was variable and ranged from 3 days after trauma to 5 years. Color Doppler ultrasound and arteriographic examinations permitted a fast and exact diagnosis and were the most commonly recommended and usually demonstrate arterial pulsations within an anechoic space in proximity to an injured vessel. Arteriography can also be useful for the evaluation of distal extremity irrigation to help decide the treatment option [1, 3, 4, 9]. In this case, arteriography would only be indicated if both pedal pulses could not be palpated [11]. The management is surgical in order to prevent possible complications such as rupture, neurologic alterations due to compression, or motor alterations such as restriction of the dorsiflexion of the foot. The technique will depend on the vascular anatomy and the patient characteristics. For those patients with a preserved irrigation via posterior tibial artery and plantar arch, suture ligation of the dorsal artery and resection of the pseudoaneurism is a safe option [3, 12]. In patients with a low blood flow (i.e., Diabetics, hypertense, etc.) or an occluded posterior tibial artery, vascular reconstruction is recommended [4, 6, 7, 12].
